# Early monitoring of plasma KRAS G12C with digital PCR predicts antitumor response to immunotherapy or sotorasib in advanced NSCLC: A brief report

**DOI:** 10.1016/j.jlb.2024.100161

**Published:** 2024-06-21

**Authors:** Andrea De Giglio, Federico Zacchini, Giulia Venturi, Alessandro Di Federico, Claudia Parisi, Filippo Gustavo Dall’Olio, Ilaria Ricciotti, Valentina Favorito, Ambrogio Gagliano, Dario De Biase, Thais Maloberti, Annalisa Altimari, Elisa Gruppioni, Giovanni Tallini, Barbara Melotti, Francesca Sperandi, Francesco Gelsomino, Lorenzo Montanaro, Andrea Ardizzoni

**Affiliations:** a Department of Medical and Surgical Sciences, University of Bologna, Bologna, Italy; b Medical Oncology, IRCCS Azienda Ospedaliero-Universitaria di Bologna, Bologna, Italy; c Departmental Program in Laboratory Medicine, IRCCS Azienda Ospedaliero-Universitaria di Bologna, Bologna, Italy; d Department of Medical Oncology, Gustave Roussy, Villejuif, France; e Faculty of Medicine, Paris-Saclay University, Paris, France; f Solid Tumor Molecular Pathology Laboratory, IRCCS Azienda Ospedaliero-Universitaria di Bologna, Bologna, Italy; g Department of Pharmacy and Biotechnology, University of Bologna, Bologna, Italy

**Keywords:** Non-small cell lung cancer, Liquid biopsy, Immunotherapy, Kras

## Abstract

**Background:**

The frontline management of non-oncogene addicted non-small cell lung cancer (NSCLC) involves immune-checkpoint inhibitors (ICI) alone or combined with chemotherapy (CT-ICI). The dynamic landscape of KRAS-positive NSCLC presents a spectrum of treatment options, including ICIs, targeted therapy and combination strategies currently under investigation.

**Methods:**

We conducted a prospective project to detect circulating tumor DNA (ctDNA) in patients with KRAS G12C, advanced NSCLC. We included patients undergoing upfront ICIs or subsequent line sotorasib. We planned three-time points: baseline (T0), after 3 months of treatment (T1) and at disease progression (T2).

**Results:**

24 consecutive patients have been included. The most frequent baseline characteristics were: nonsquamous histology (95.8%), male gender (62.5%), ECOG PS 0–1 (79.2%), <3 metastatic sites (13/24, 54.2%). 18 patients (75%) received ICI-based strategies and 6 patients (25%) sotorasib. Patients with liver metastases (p ​= ​0.01) and those with >3 metastatic sites (p ​= ​0.002) exhibited significantly elevated ctDNA. Median overall survival (OS) was 7.5 months, progression-free survival (PFS) was 4.0 months and the objective response rate (ORR) was 33.3%. Higher AF correlated with an increased risk of death (HR 1.04, p ​= ​0.03), though not progression. The mOS was 7.5 months (95% CI, 1.91-NR) in high-AF group and 11.3 months (95% CI, 6.6-NR) in low-AF group (p ​= ​0.38). Notably, a reduction in plasma DNA levels was significantly associated with objective response (p ​= ​0.01). Two patients received a T2 dosage showing increased ctDNA levels after a previous reduction associated with response.

**Conclusion:**

Early monitoring with ctDNA may offer potential benefits in the evolving scenario of KRAS G12C NSCLC treatment.

## 1 Introduction

The current first-line therapy for advanced non-oncogene addicted non-small cell lung cancer (NSCLC) involves immune checkpoint inhibitors (ICIs) as single agents or combined with chemotherapy or other ICIs [1]. Notably, the objective response rates do not exceed 45%, and up to 30% of patients experience primary resistance or early death to treatment [1].

Approximately 30 % of lung adenocarcinomas harbor an activating mutation in the KRAS gene, historically an ‘orphan' of targeted therapy [2]. Recently, a revolution involving the KRAS-positive NSCLC has led to a growing armamentarium of therapeutics ranging from immunotherapy to targeted therapy and combination strategies currently under evaluation [2]. Phase 1–2 studies have documented for the first time the efficacy of the KRAS p.G12C mutation inhibitors sotorasib and, more recently, adagrasib in pretreated patients [3,4]. Furthermore, in the Codebreak200 phase 3 randomized trial, sotorasib exhibited superior clinical efficacy over docetaxel in KRAS G12C patients who experienced progression to ICIs-based treatments [5].

As therapeutic options expand and prognostic biomarkers emerge, optimizing NSCLC genotyping becomes crucial to enhance patient care and economic sustainability.

Liquid biopsy is a minimally invasive technique employed to identify mutations in circulating tumor DNA (ctDNA), offering an alternative means of detecting tumor genomic alterations with a growing role in NSCLC management [6]. Although the technique boasts a high degree of specificity, sensitivity levels are less satisfying, with a maximum of 70% [6]. Allele-specific amplification and emulsion polymerase chain reaction (PCR) assays are utilized in PCR-based platforms to identify mutant DNA, which may take up to 3 days to produce outcomes [6]. Conversely, plasma-testing platforms based on next-generation sequencing (NGS) may have a turnaround time of up to two weeks [7].

Monitoring of *KRAS* mutations in ctDNA using PCR aimed at allele frequency (AF) determination of mutant alleles (e.g. G12C) may find application in the dynamic evaluation of therapeutic response [8].

In the present study, we report the outcomes of a translational project designed to offer early monitoring with ctDNA in advanced NSCLC patients undergoing upfront immunotherapy-based strategies or sotorasib.

## 2 Materials and method

We conducted an academic, prospective, observational study with translational analysis between November 2021 and August 2023 ​at the IRCCS Azienda Ospedaliera Universitaria of Bologna, Italy. The population consisted of 24 patients with advanced *KRAS* G12C mutant NSCLC eligible for first-line immunotherapy-based treatments or subsequent lines of KRAS inhibitors. The primary objective was to establish the association of plasma variation of *KRAS* G12C ctDNA with treatment response and survival outcomes. The secondary objective was to analyze the prognostic impact of baseline AF levels of the *KRAS* mutation detected on ctDNA. We performed the dosage of *KRAS* mutant alleles on circulating DNA with digital droplet PCR (ddPCR) at three preplanned time points: baseline (T0), after 3 months of treatment (T1), and at disease progression (T2). Laboratory methods were resumed in the supplementary materials.

### 2.1 Laboratory methods

Blood samples (10 ​mL) were collected in EDTA tubes at specific time points and processed within 3 ​h. Blood was centrifuged at 2000 g for 10 ​min at 4 ​°C to separate plasma. The obtained plasma was stored at −80 ​°C. Circulating free DNA was then extracted from 4 ​ml of plasma with QIAamp Circulating Nucleic Acid Kit (Qiagen) in line with manufacturer's instructions and stored at −20 ​°C. A specific kit from Bio-Rad (ddPCR mut assay KRAS p.G12C.34 ​GT Cat. 10049550) was acquired for detecting and quantifying wild-type (wt) and p.G12C mutated KRAS (specific for 34 ​G ​> ​T). The samples were partitioned into a mean of 15,000 droplets by using QX200 Droplet Generation (Bio-Rad). Then, PCR reaction was prepared according to the manufacturer's instructions and the droplets were analyzed using the QX200 Droplet Reader (Bio-Rad), to provide absolute quantification of wt or mutated KRAS. The results were analyzed with the QuantaSoft Analysis Pro Software (v1.0 Bio-Rad). A cut-off of three droplets was used to define a sample as mutant, according to Poisson's law of small numbers (as reported in the manufacturer's instructions). According to this limit, we could detect a minimum amount of 2.7 copies per ml of plasma. The mutant allele fraction (AF) was calculated as the number of mutated droplets/(wt ​+ ​mutated droplets). The kit used had a detection limit of AF of 0.2% as reported in the manufacturer's instructions.

### 2.2 Statistical methods

The clinical and laboratory findings were presented as continuous and categorical variables and median values and proportions were used to summarize them. Means and proportions were compared by performing T-test (Pearson correlation test or Kruskal-Wallis test if required) and chi2-test (or Fisher's exact test if required). The overall survival (OS) was defined as the duration between treatment start and death due to any cause, while progression-free survival (PFS) was defined as the duration between treatment start and radiological or clinical progression or death due to any cause. The objective response rate (ORR) was expressed as the percentage of patients who achieved a partial or complete response according to the RECIST 1.1 criteria as assessed by physicians. The Kaplan-Meier method was used to estimate OS and the Log-rank Test was used to compare OS curves according to score prognostic assessment. The relationship between variables and survival outcomes was explored through Cox model regression. A p-value ≤0.05 was considered statistically significant.

## 3 Results

Twenty-four consecutive patients were enrolled. The most frequent baseline characteristics were: nonsquamous histology (23/24, 95.8%), male gender (15/24,62.5%), ECOG PS 0–1 (19/24, 79.2%), less than three metastatic sites (13/24, 54.2%). Thirteen patients (54.2%) received histology-driven CT-ICI, 5 (20.8%) received ICI single-agent and 6 patients (25%) were treated with sotorasib in a subsequent line (Table 1).

**Table 1:** Baseline characteristics.

		Total (%)
Age	<70	14 (58.3)
	≥70	10 (41.7)
Histology	nonsquamous	23 (95.8)
	squamous	1 (4.2)
Sex	female	9 (37.5)
	male	15 (62.5)
ECOG PS	<2	19 (79.2)
	≥2	5 (20.8)
Number of met. sites	<3	13 (54.2)
	≥3	11 (45.8)
Brain met.	no	16 (66.7)
	yes	8 (33.3)
Liver met.	no	20 (83.3)
	yes	4 (16.7)
Lung met.	no	10 (41.7)
	yes	14 (58.3)
Bone met.	no	13 (54.2)
	yes	11 (45.8)
Line of treatment	first	18 (75)
	subsequent	6 (25)
Type of treatment	chemo-immunotherapy	13 (54.2)
	immunotherapy	5 (20.8)
	sotorasib	6 (25.0)

**Fig. 1:**
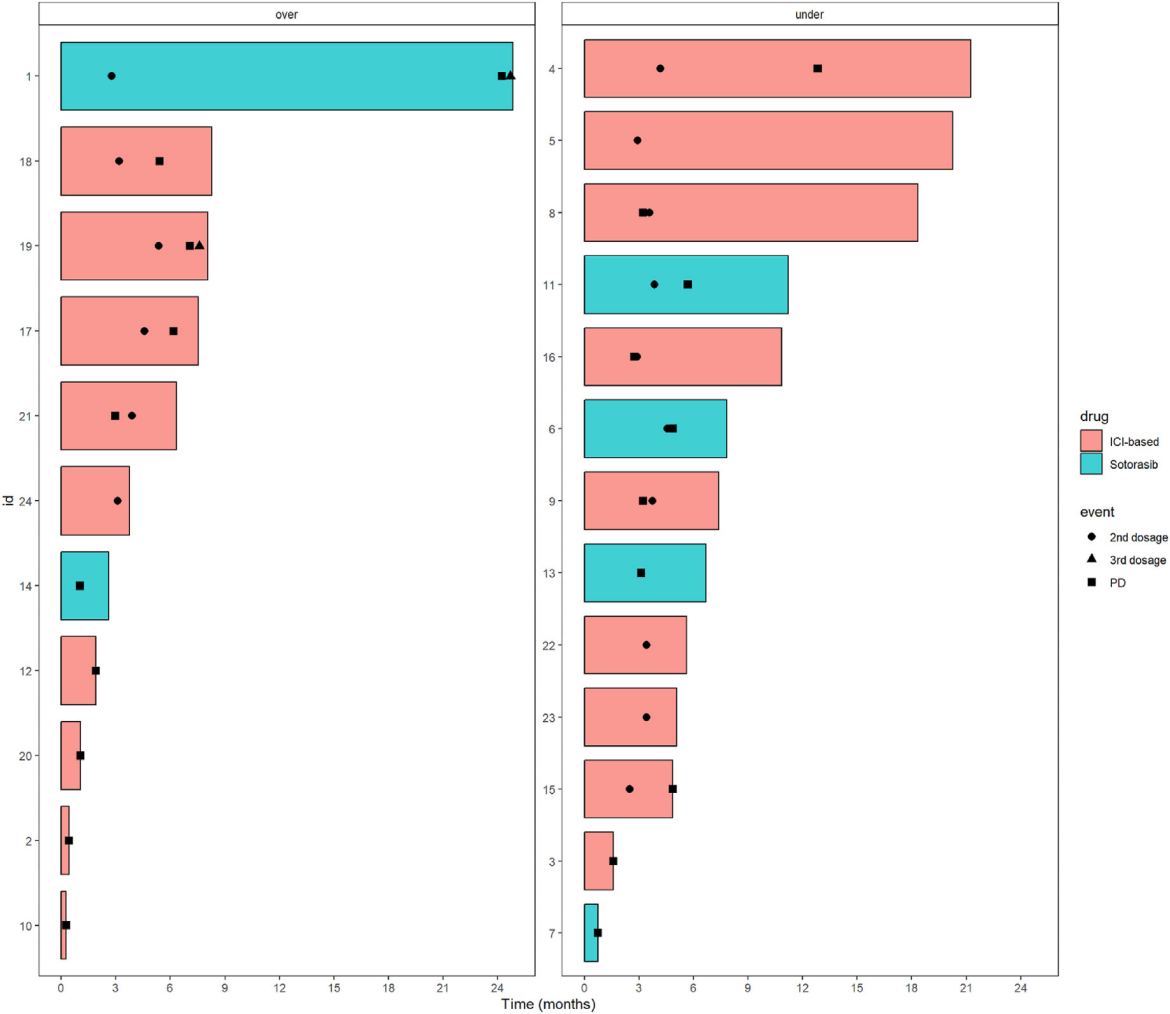
Swimmer plot of patients enrolled in the prospective cohort according to treatment received and level of baseline Allele Frequency (over vs. under median AF).

**Fig. 2:**
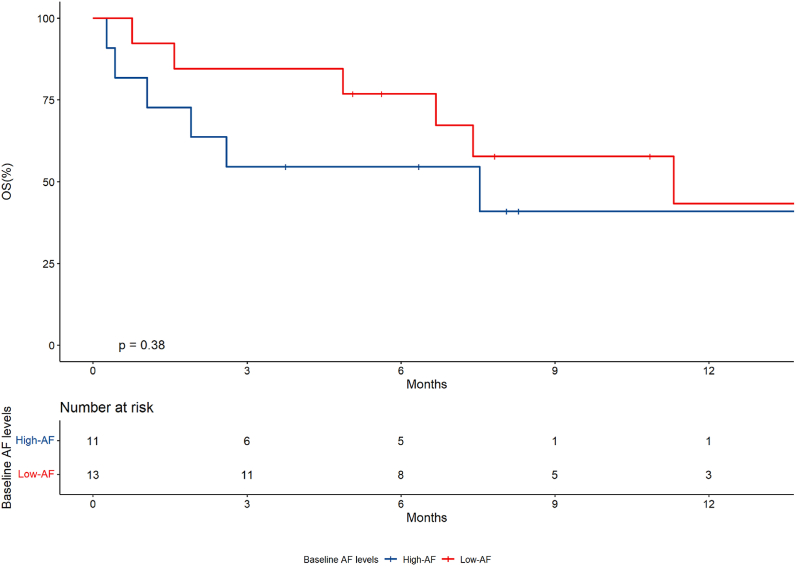
Overall survival according to baseline KRAS G12C allele frequency (AF).

Seventeen of 24 patients (70.8%) had detectable circulating DNA at T0 dosage. The median plasma DNA concentration was 6.81 cp/ml (IQR 0–56.7). Patients with baseline liver metastases (4/24, 16.7%) had a significantly increased mean T0 DNA concentration (1157.4 cp/ml) in comparison with patients without them (27.8 cp/ml) (p 0.01) (Fig. 1S). The median T0 AF was 1.2 % (IQR, 0–9.9). The presence of at least 3 metastatic sites significantly increased mean T0 AF (17.4%) compared to 1–2 metastatic sites (1.2%) (p 0.002) (Fig. 2S).

Sixteen of 24 patients (66.6%) underwent T1 dosage after three months of treatment per protocol. Six out of 24 patients (25%) died within 90 days from treatment start, one patient did not receive T1 dosage due to worsening clinical conditions and being discharged to home and one patient refused to undergo T1 dosage. The mean plasma DNA concentration was 19.7 cp/ml (IQR 0–21.2) at T1. Four of 16 patients (25%) had increased plasma DNA levels compared to T0. Of them, 2 patients had undetectable plasma DNA at T0. Eight of 16 patients (50%) presented decreased DNA levels compared to T0, and 5 of them had a complete plasma clearance at T1. 4/16 patients (25%) had undetectable plasma DNA both at T0 and T1. The median T1 AF was 0.2% (IQR 0–6.0).

Two patients underwent liquid biopsy at three-time points. In both cases, we registered a decrease in plasma DNA level at T1 (id 1, 11.8 cp/ml to not detectable; id 19, 50 cp/ml to 5.8 cp/ml) with a concomitant radiological partial response. At T2, according to the protocol for progressive disease (PD) recognition, we observed in both cases an increase in DNA levels (id 1, 13.7 cp/ml; id 19, 19 cp/ml) (Fig. 1).

The median overall survival (OS) was 7.5 months (95% CI, 4.8-NR), and the median progression-free survival (PFS) was 4.0 months (95% CI, 2.9–7.0).

When analyzed as a continuous variable, baseline AF was associated with an increased risk of death (HR 1.04, 95% CI, 1.0–1.08, p ​= ​0.03) but not progression (HR 1.02, 95% CI, 0.97–1.06, p ​= ​0.1) in the univariate regression model.

We used the median T0 AF (1.2%) to dichotomize patients in high-AF and low-AF groups. The median OS was 7.5 months (95% CI, 1.91-NR) in the high-AF group and 11.3 months (95% CI, 6.6-NR) in the low-AF group (p ​= ​0.38) (Fig. 2). The median PFS was 2.9 months (95% CI, 1.05 – NR) and 4.8 months (95% CI, 3.1-NR), respectively in the high-AF and low-AF groups (p ​= ​0.58).

The ORR was 33.3% (95% CI, 17%–53%). The mean T0 plasma DNA levels were 171.7 cp/ml among responders and 258.2 cp/ml among non-responders (p ​= ​0.36) (Fig. 3S). The mean T0 AF was 10.9 among responders and 8.3 among non-responders (p ​= ​0.27) (Fig. 4S). A decrease in plasma DNA levels from T0 to T1 was significantly associated with objective response (p ​= ​0.01).

## 4 Discussion

In our single-center, translational study, we demonstrated the valuable role of early longitudinal *KRAS* G12C blood monitoring in accurately predicting the response to upfront immunotherapy or sotorasib. Our analyses confirmed the research hypotheses, corroborating continuous baseline AF as a predictor of death risk and early longitudinal changes of ctDNA as predictors of anti-tumor response.

The prognostic evaluation of AF from baseline ctDNA in advanced solid malignancies has been investigated in several studies. In a prospective investigation including BRAF-V600E advanced colorectal patients, the prognostic role of plasma AF was explored through digital-droplet PCR [9]. Patients with higher baseline AF (2% threshold) reported significantly decreased PFS and OS. A phase 2 clinical trial was conducted to study the effectiveness of Pembrolizumab in advanced solid tumors, including a group of patients with NSCLC [10]. The trial also explored the potentiality as predictive tool of personalized ctDNA analysis based on tissue whole exome sequencing findings [10]. The authors found that lower ctDNA levels at baseline were associated with improved survival outcomes (OS, PFS) [10].

Remarkably, the EGFR-positive disease may be considered the paradigm of oncogene-addicted NSCLC, for which the AF detected at baseline or during targeted therapy represents a clear predictor of response and survival outcomes [11,12]. The present findings support the utilization of AF ctDNA as a baseline prognostic factor, which serves as a surrogate marker of disease burden or aggressiveness.

The variation of ctDNA levels has been related to disease response or progression in our cohort. The previously discussed study by Bratman et al. also demonstrated that ctDNA kinetics strongly correlated with treatment response and, particularly, that ctDNA clearance was associated with long-term benefit from Pembrolizumab [10]. Another single-center study prospectively enrolled 58 patients affected by *KRAS* positive advanced NSCLC and treated with single-agent immunotherapy or chemotherapy for whom frozen plasma samples were available at two time points (baseline vs 3 or 4 weeks) [13]. Liquid biopsy was performed through a ddPCR, finding a correlation between survival outcomes and early ctDNA variations [13].

Analogously, Ricciuti et al. investigated the role of ctDNA changes in 62 NSCLC patients treated with upfront Pembrolizumab, either as a single agent or in combination with platinum doublets [14]. Tumor response and longer survival outcomes were significantly associated with plasma AF decrease at the first timepoint (median of 21 days from therapy start). Recently, Paweletz et al. conducted an exploratory analysis of ctDNA changes in a phase 2 trial evaluating adagrasib activity in *KRAS* G12C advanced NSCLC [15]. Interestingly, plasma samples were analyzed with NGS and ddPCR at longitudinal time points, and the results were presented as AF. The authors found an excellent correlation between NGS and ddPCR analysis, paving the way for using the latter as a faster and cheaper plasma analysis for ctDNA in KRAS-positive NSCLC. Notably, the clearance of ctDNA at cycles 2 and 4 of treatment was associated with improved ORR and survival outcomes (OS, PFS), respectively. In our analysis, we proposed a ctDNA analysis performed using ddPCR at baseline and after 4 cycles of treatment (3 months), demonstrating a significant association with tumor response.

The present work's main limitation is a restricted sample size with a short follow-up time. However, our research, consistent with prior studies, points out that plasma ddPCR can serve as a dynamic method for monitoring treatment efficacy, playing an essential role in identifying early treatment failure in conjunction with radiological findings. In plenty of competitive treatments, ddPCR plasma genotyping can help adjust treatment intensity to reduce toxicity and prevent failure.

## Funding

This work was supported by Fondazione Carisbo (grant 2021 – ID 19320); the IRCCS Azienda Ospedaliero-Universitaria di Bologna, Research Funding (WFR RC-2022-2773346, project RC22000505)

## CrediT author statement

Andrea De Giglio: Conceptualization, Methodology, Writing- Original draft preparation. Federico Zacchini: Investigation, Resources, Data curation, Writing- Reviewing and Editing, Giulia Venturi: Investigation, Resources, Data curation, Writing- Reviewing and Editing. Alessandro Di Federico: Writing- Reviewing and Editing. Claudia Parisi: Methodology, Writing- Reviewing and Editing. Filippo Gustavo Dall’Olio: Writing- Reviewing and Editing. Ilaria Ricciotti: data curation, Writing- Reviewing and Editing. Ambrogio Gagliano: data curation, Writing- Reviewing and Editing.Valentina Favorito: data curation, Writing- Reviewing and Editing. Dario De Biase: Writing- Reviewing and Editing. Thais Maloberti: Writing- Reviewing and Editing. Annalisa Altimari: Writing- Reviewing and Editing. Elisa Gruppioni: Writing- Reviewing and Editing. Giovanni Tallini: Writing-Reviewing and Editing. Barbara Melotti: Writing- Reviewing and Editing. Francesca Sperandi: Writing- Reviewing and Editing. Francesco Gelsomino: Writing- Reviewing and Editing. Lorenzo Montanaro: Funding acquisition, supervision, Writing- Reviewing and Editing. Andrea Ardizzoni: supervision, project administration, Writing- Reviewing and Editing.

## Ethics approval and patient consent

This study was conducted following the principles of the Declaration of Helsinki (1964) after receiving approval from the local Ethics Committee (Comitato Etico Area Vasta Emilia Centro, approval no.783/2021/Sper/AOUBo). Written informed consent was obtained from all patients.

## Declaration of competing interest

The authors declare the following financial interests/personal relationships which may be considered as potential competing interests:

Francesco Gelsomino reports a relationship with Eli-Lilly, Novartis, AstraZeneca, Bristol-Meyers that includes: consulting or advisory. Alessandro Di Federico reports a relationship with Society for Immunotherapy of Cancer, Hansen-Wade. that includes: consulting or advisory and travel reimbursement. Andrea Ardizzoni reports a relationship with BMS, Eli-Lilly, MSD, AZ, 10.13039/100004337Roche, Takeda, Janssen, 10.13039/100004339Sanofi, 10.13039/100004336Novartis, 10.13039/100006483AbbVie, Daiichi that includes: consulting or advisory and speaking and lecture fees. If there are other authors, they declare that they have no known competing financial interests or personal relationships that could have appeared to influence the work reported in this paper.
