# Supplementary material for: Early monitoring of plasma KRAS G12C with digital PCR predicts antitumor response to immunotherapy or sotorasib in advanced NSCLC: A brief report
Source: J Liq Biopsy. 2024 Jun 21;6:100161. doi: 10.1016/j.jlb.2024.100161 (PMC11863973; doi:10.1016/j.jlb.2024.100161)
Supplement: Multimedia component 1 [file mmc1.docx]

**Supplementary**


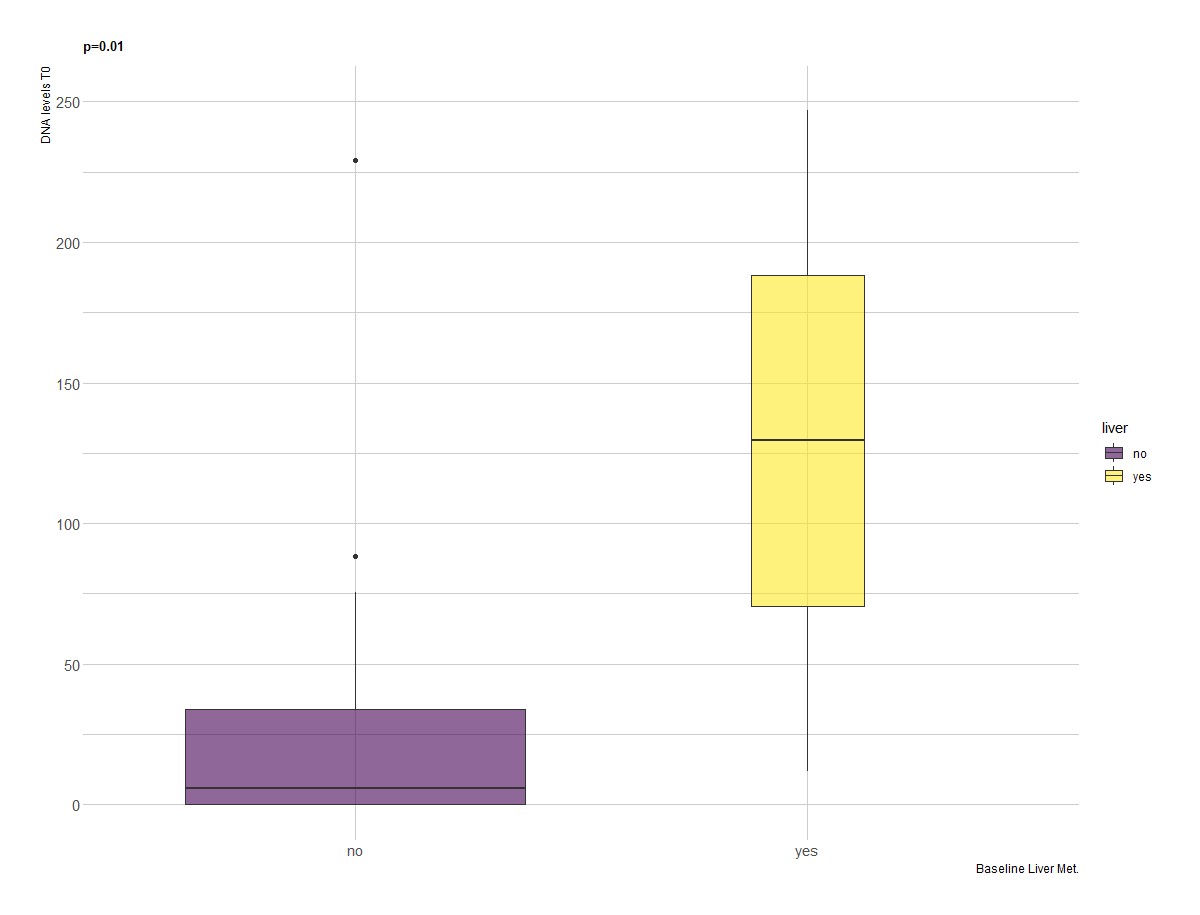


**Figure 1S. Plasma DNA levels (cp/ml) at T0 according to the presence of baseline liver metastasis.**


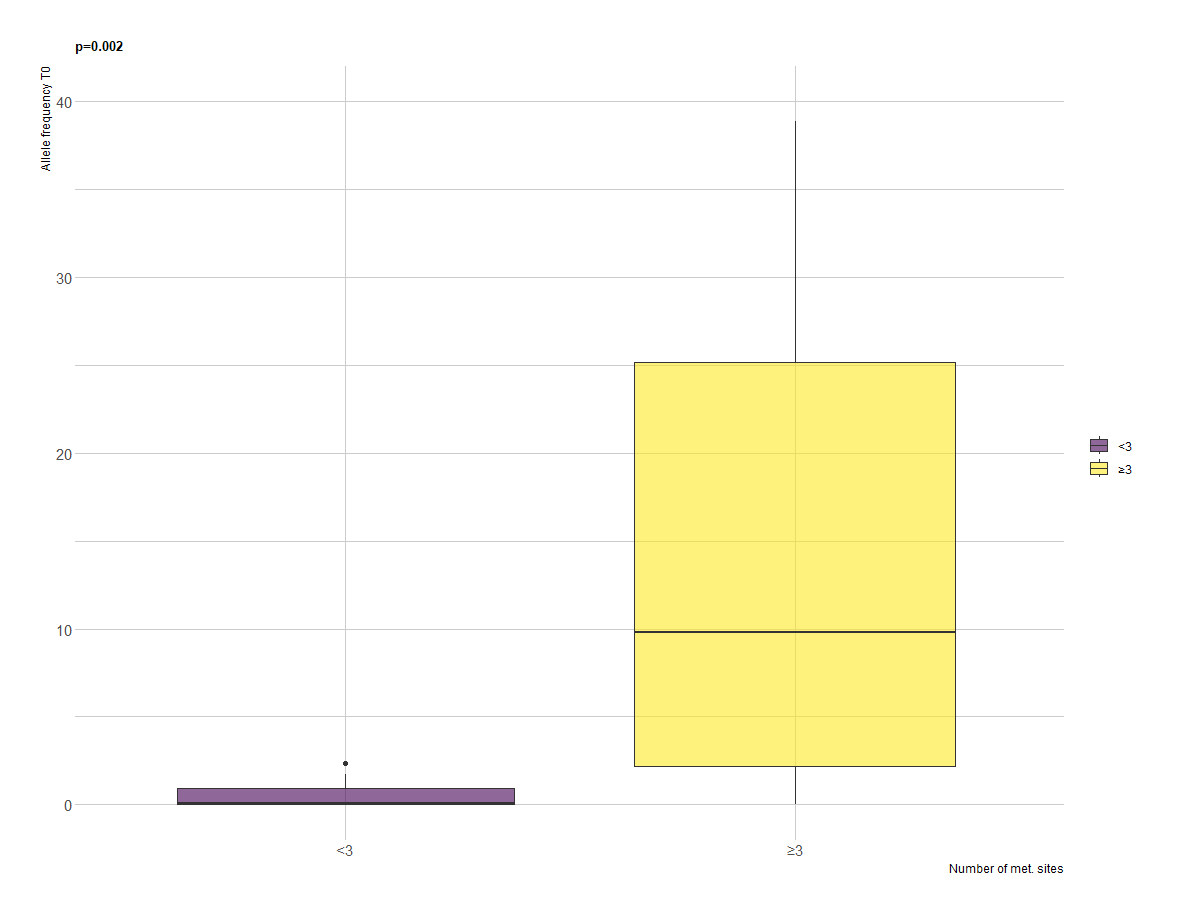


**Figure 2S. Plasma allele frequency (AF) at T0 according to the baseline number of metastatic sites.**


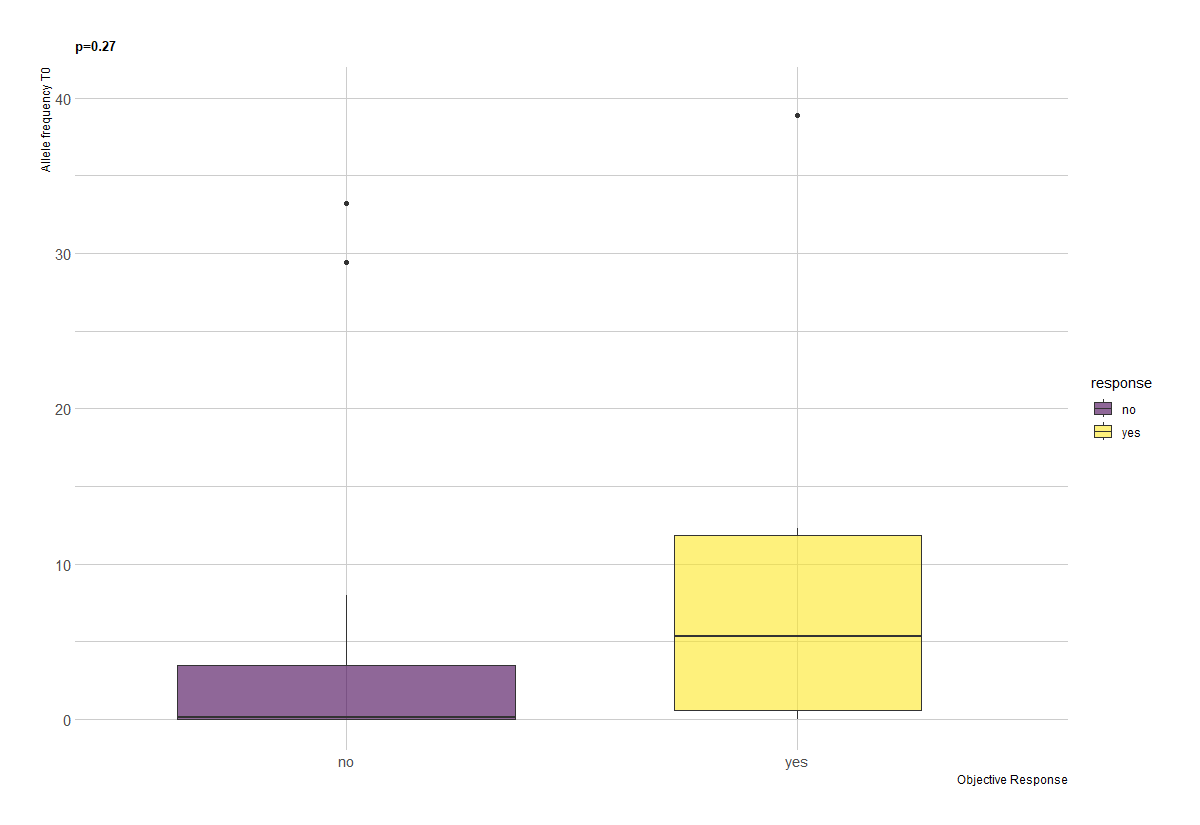


**Figure 3S. Plasma allele frequency (AF) at T0 according to objective response**


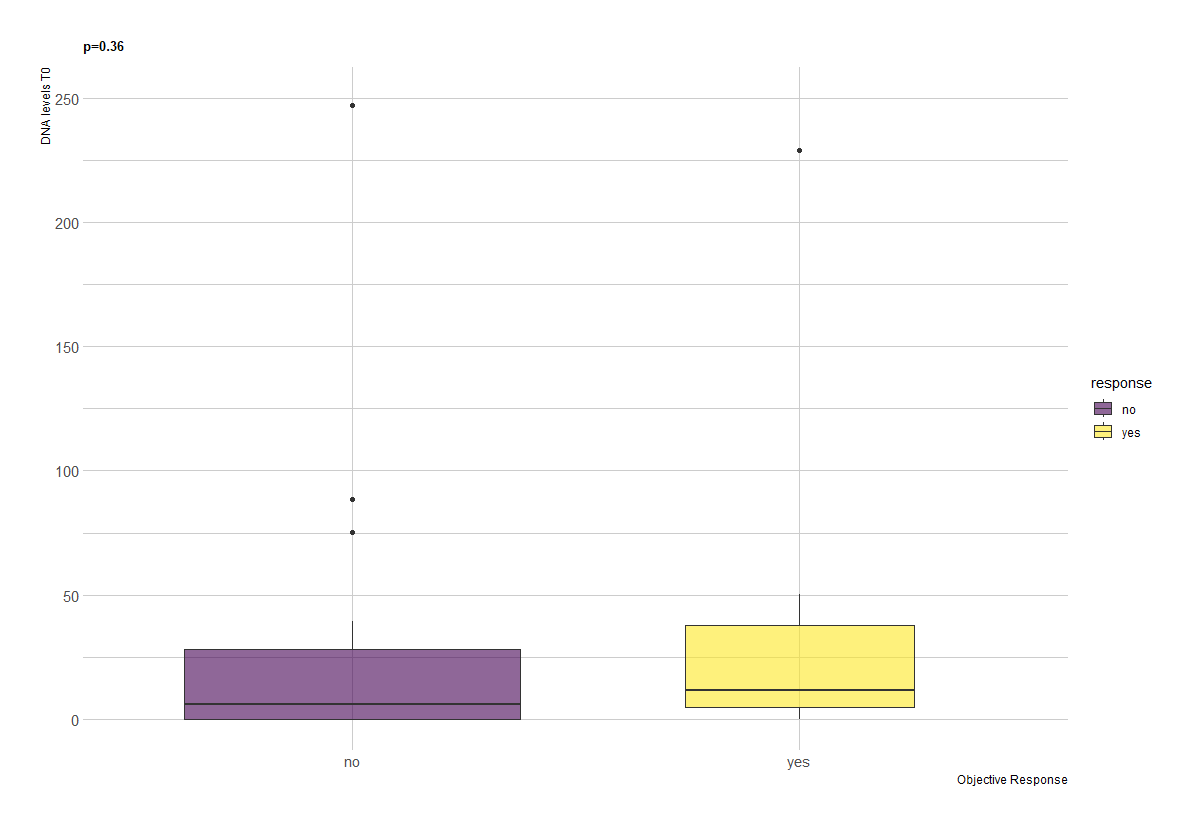


**Figure 4S. Plasma DNA levels (cp/ml) at T0 according to objective response**
